# Observational Study on Subcutaneous Immunotherapy for Japanese Cedar Pollinosis in 2024: Comparison With 2019

**DOI:** 10.1002/iid3.70589

**Published:** 2026-11-13

**Authors:** Otsuka Hirokuni, Okubo Kimihiro, Otsuka Kuninori, Gotoh Minoru

**Affiliations:** ^1^ Otsuka ENT Clinic Otorhinolaryngology Kanagawa Japan; ^2^ Musashikosugi Hospital, Otorhinolaryngology Nippon Medical School Kanagawa Japan; ^3^ Otorhinolaryngology and Head and Neck Surgery Nippon Medical School Kanagawa Japan

**Keywords:** eosinophil, Japanese cedar pollinosis, mast cell, metachromatic cell, nasal surface, nasal swabs, neutrophil, observational cohorts, subcutaneous immunotherapy, sublingual immunotherapy

## Abstract

**Background and Objective:**

In a 2019 observational cohort study, we showed that subcutaneous allergen immunotherapy (SCIT) reduced symptoms in the preseason and during the Japanese cedar pollinosis (JCP) season. SCIT reduced metachromatic cells (Mc) and eosinophils in nasal swabs in subjects with JCP. The current 2024 study is compared with our 2019 study, focusing on the two JCP seasons to determine whether SCIT could further reduce symptoms and the counts of these cells in nasal swabs, particularly in patients who had received SCIT for ≥ 5 years.

**Methods:**

Thirty‐three subjects with SCIT treatment for JCP and 53 subjects without SCIT in the 2019 season, and 30 subjects with SCIT treatment and 40 subjects without SCIT in the 2024 season, were interviewed in season in both years, and Mc and eosinophil counts were examined in nasal swabs.

**Results:**

In 2019, there was a significant difference between the 33 subject visits with SCIT and the 53 subjects without SCIT: *p* = 2.2 × 10^−5^ for severity, *p* = 0.002 for Mc counts in nasal swabs, and *p* = 0.008 for eosinophil counts. In 2024, there was a significant difference between the 30 subject visits with SCIT and the 40 subjects without SCIT: *p* = 2.4 × 10^−8^ for severity, *p *= 2.6 × 10^−6^ for Mc counts in nasal swabs, and *p* = 2.7 × 10^−8^ for eosinophil counts. The 13 cases investigated in both 2019 and 2024 that had been on SCIT continuously showed significantly improved outcomes in 2024, in comparison to those on shorter‐duration SCIT treatment.

**Conclusion:**

SCIT can be safely administered at the doses described below, and a 2024 study showed not only symptom relief but also a dramatic reduction in Mc and eosinophil counts in nasal swabs compared to a 2019 study.

## 1 Introduction

Allergen immunotherapy has long been considered a fundamental treatment for airway allergies, such as allergic rhinitis and bronchial asthma, that cannot be achieved through drug therapy. In the past, subcutaneous immunotherapy (SCIT) was used, but recently, more people are opting for sublingual immunotherapy (SLIT) [1, 2, 3]. This is because allergen subcutaneous immunotherapy can cause side effects such as local reactions, systemic pruritus, respiratory distress, and rarely, death after treatment [4, 5, 6]. Thus, because sublingual immunotherapy has fewer side effects, it is safer and recommended. However, SCIT for JCP is safe and sufficiently effective if the dose of allergen immunotherapy is carefully titered so the diameter of the skin reactive wheal is ≤20–25 mm 15 min after subcutaneous injection [7]. Furthermore, at this dosage, symptom improvement was observed, along with a decrease in the numbers of metachromatic cells (Mc; mast cells and basophils) and eosinophils in nasal swabs [7]. Five years later, in 2024, we confirmed that SCIT was safe for JCP, and showed that individuals on SCIT for ≥ 5 years had a further decrease in the number of Mc and eosinophils in nasal swabs.

### 1.1 Pollen Count

As previously reported [7], Japanese cedar pollen counts were measured in Yokohama using the Durham method [8]. A significant pollen count for that year was defined as ≥ 10 pollen grains/cm^2^ or ≥ 20 pollen grains/cm^2^/day for ≥ 2 consecutive days, ending with < 10 pollen grains/cm^2^. In 2019, these counts were February 19 and April 6, whereas in 2024, these counts were February 15 and April 6, respectively. The cumulative Japanese cedar pollen counts/cm^2^ in Yokohama were 3696 in 2019 and 4432 in 2024.

### 1.2 Subjects

The studies were conducted during the Japanese cedar pollen seasons in 2019 and 2024. All subjects were previously diagnosed with JCP according to Japanese Guidelines and had typical symptoms and eosinophilia in nasal swabs [9]. They were positive for IgE antibodies to Japanese cedar pollen with class 1 or more reactivity. Subjects positive for IgE antibodies to mite under class 1 and without perennial symptoms, and subjects with IgE antibodies to orchard grass, but with no symptoms in orchard season, were included. Subjects were negative for IgE antibodies to ragweed, mugwort, and Alternaria. All participants provided informed consent. The study was approved by the Ethics Committee of Nippon Medical School.

In 2019, depending on the preference of the subjects with JCP who attended our clinic, they were given SCIT (*n* = 33) or not (*n* = 53) and were compared for symptoms, Mc, eosinophil counts, and neutrophil counts during the pollen season [7]. In 2024, subjects with JCP were given SCIT (*n *= 30) or not (*n *= 40) and were analyzed as done in 2019 (Table 1). Subjects with SCIT visited the clinic monthly during the pollen season, whereas those without SCIT visited as needed.

**Table 1:** Characteristics of subjects in the subcutaneous immunotherapy and untreated groups in 2019 and 2024.

	2019		*p* value^a^	2024		*p* value^a^
	SCIT	Non‐SCIT		SCIT	Non‐SCIT	
Number	33	53		30	40	
Age ± SE	55.0 ± 1.8	50.1 ± 1.9	0.1	50.6 ± 2.8	48.1 ± 3.0	0.41
	(34–74)	(28–81)		(18–70)	(17–84)	
M:F	10:23	22:31	0.41	14:16	19:21	0.19

*Note:* In 2019 and 2024, no significant differences in age and gender were observed between the SCIT and non‐SCIT.

^a^ *p* value among SCIT and non‐SCIT groups.

The analyses of their symptoms and cytology were conducted on the results from their first visits in 2019 and 2024. In both 2019 and 2024, 53 and 40 non‐SCIT subjects were those who visited the clinic for the first time to receive preventive and symptomatic medication during the season.

## 2 Methods

### 2.1 Comparison of Degree of Nasal Symptom Severity, Metachromatic Cells, and Eosinophils in Patients Treated With SCIT and Without SCIT in 2019 and 2024

To examine whether SCIT treatment changed clinical symptoms, Mc and eosinophil counts in nasal swabs in 2019 and 2024, 51 subject visits who underwent SCIT were compared with 53 subjects who did not undergo SCIT in 2019, and 43 subject visits who underwent SCIT were compared with 40 subjects who did not undergo SCIT in 2024. The *p* values for 2019 and 2024 were then compared using the Mann–Whitney U test.

### 2.2 Subjects' Symptoms and Severity Grading

All subjects completed a detailed symptoms questionnaire at each visit [7]. Classification of symptom severity used the Japanese Guidelines [9]. Severity grading was classified as: most severe, severe, moderate, mild, and no symptoms.

### 2.3 Analysis of Cell Types in Nasal Swabs

Cells in swabs were assessed as described previously [7]. The numbers of neutrophils and eosinophils were graded as: −; none, ±: few scattered cells, 1+; found cells easily, 2+; abundant cells, with small clumps, 3+; abundant cells, often in clumps. The numbers of Mc on the entire slide were classified as: 0, 1–9 cells, 10–99 cells, 100–999 cells, ≥ 1000 cells. These cell counts were analyzed in a blinded manner so that the subject source of the samples was not known to the evaluator.

### 2.4 Statistical Analyses

For comparison of groups, a *t*‐test for age, a *χ* ^2^ test for sex ratio, and the Mann–Whitney U test for symptom severity and cell numbers were used. *p* < 0.05 was considered statistically significant.

## 3 Results

### 3.1 Subcutaneous Immunotherapy, Safety, and Optimization of Maintenance Doses

When we commenced SCIT in our clinic in 1998, the original protocol used for SCIT was based on the manual of SCIT used by the Nippon Medical School (Table 2). However, side effects such as redness with itching of the face, arms and legs or whole body, and wheezing appeared in some patients after allergen injections. At that time, the maintenance dose for SCIT was determined when the local skin redness reaction was < 50 mm after 15 min. In all patients who experienced side effects, the swelling site (not redness) was > 40 mm 15 min after the injection. Given these side effects, in 2006 we began to use a maintenance dose based on 20–25 mm of swelling at the skin injection site. No subject reported significant side effects or anaphylaxis using this latter approach.

**Table 2:** Schedule of subcutaneous immunotherapy.

Intradermal injection was started and continued twice a week as below.						
0.2 JAU/mL	0.05, 0.07, 0.1, 0.15, 0.2, 0.3 mL					
2 JAU/mL	0.05, 0.07, 0.1, 0.15, 0.2, 0.3 mL					
20 JAU/mL	0.05, 0.07, 0.1, 0.15, 0.2, 0.3 mL					
200 JAU/mL	0.05, 0.07, 0.1, 0.15, 0.2, 0.3 mL					
Maintenance amount in 33 of 2019 and 30 (2024) patients (keep the injection every month until this study is completed)						
Patients numbers	1 (2)	5 (4)	8 (6)	5 (7)	4 (4)	1 (1)
20 JAU, mL	0.05	0.07	0.1	0.15	0.2	0.3
Patients numbers	0 (0)	2 (0)	2 (0)	2 (2)	2 (3)	1 (1)
200 JAU, mL	0.05	0.07	0.1	0.15	0.2	0.3

*Note:* When the swelling at the injected skin site was between 20 and 25 mm, the same amount of extra was used to inject once a week for four times, then once every 2 weeks for four times, and then continued once a month until this study.

The subjects in the current study initially received 0.05 mL subcutaneous injections of 0.2 Japanese Allergy Units (JAU)/mL of standardized Japanese cedar pollen extract (Torii Pharmaceutical Co., Ltd., Tokyo, Japan). To determine the optimized allergen dose for each subject, the volume injected was increased as outlined in Table 2 twice weekly until a 20–25 mm diameter swelling occurred at the skin injection site by 15 min [7]. Thereafter, they received this optimized amount of extract weekly for 4 weeks and once every 2 weeks for 8 weeks and then once a month all year long, or until study completion (Table 2). Using this protocol, the subjects had limited side effects and no anaphylaxis.

Figure 1 shows the maintenance doses (JAU/injection) of patients determined during the 2019 and 2024 studies. The distributions of the optimized maintenance doses in the 2019 and 2024 studies were similar (*p *= 0.75 a Mann–Whitney U statistic). Table 3 shows the duration of years that subjects had been on SCIT treatment in the 2019 and 2024 seasons.

**Figure 1:**
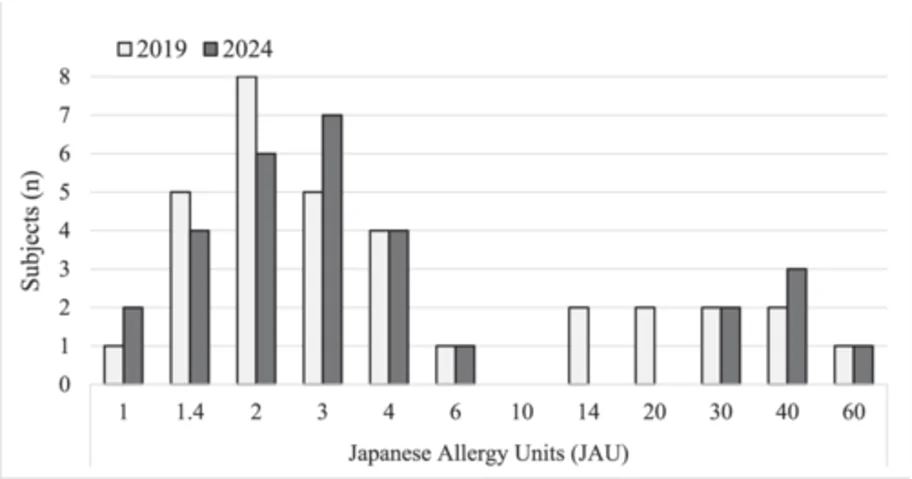
Optimized maintenance doses in 33 (2019) and 30 (2024) subjects (dosage given monthly until study completion).

**Table 3:** Duration of subcutaneous immunotherapy in 33 subjects in 2019 and 30 in 2024.

2019						
Average of duration ± SE (years)		6.11 ± 0.67 (0.5–17.7)				
Duration (years)	0.5–1.0	1.1–1.9	2.0–3.9	4.0–6.9	7–9.9	10–
Patient number	4	0	5	14	6	4
2024						
Average of duration ± SE (years)		7.52 ± 0.88 (0.5–15.5)				
Duration (years)	0.5–1.0	1.1–1.9	2.0–3.9	4.0–6.9	7–9.9	10–
Patient number	4	1	2	8	7	8

### 3.2 Comparison of Severity of Symptoms Between SCIT and Untreated Subjects in 2019 and 2024

The severity of JCP in SCIT subjects in 2019 was significantly lower (2.2 × 10^−5^; Mann–Whitney U test) than in non‐SCIT subjects. In non‐SCIT subjects (*n *= 53), the combination of numbers of severe and very severe cases was 51%, compared with 10% in SCIT subject visits (*n *= 33) (Figure 2). In the 2024 study, the severity of SCIT subjects was also significantly lower (*p* = 2.4 × 10^−8^) than in non‐SCIT subjects: the combined severity of severe and very severe was 65% in non‐SCIT subjects (*n *= 40) versus 9% in SCIT subject visits (*n *= 30) (Figure 2).

**Figure 2:**
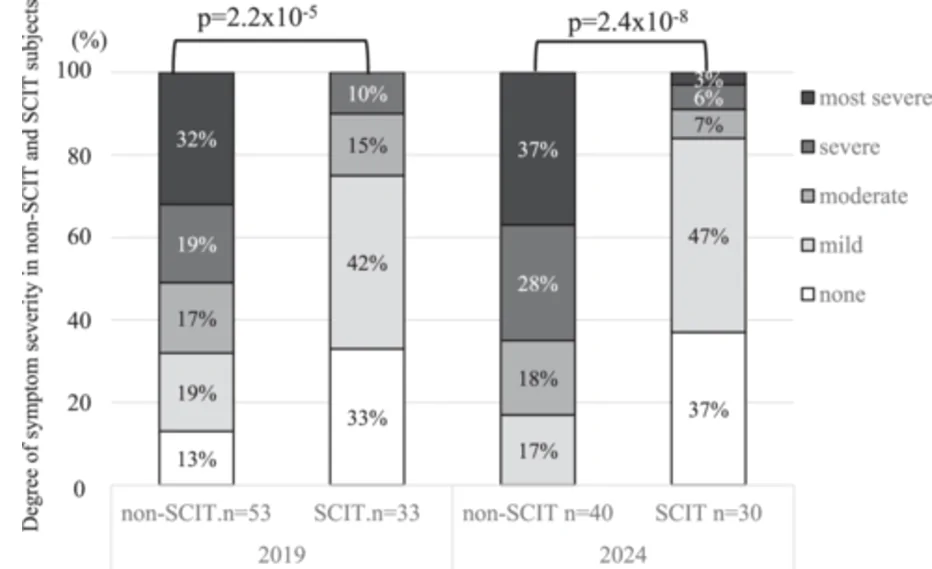
Symptom severity in non‐SCIT and SCIT subjects in 2019 and 2024. SCIT subjects had significantly lower symptom scores than non‐SCIT subjects in 2019 (*p* = 2.2 × 10^−5^) and 2024 (*p* = 2.4 × 10^−8^).

### 3.3 Nasal Swab Cytology Between SCIT and Untreated Subjects in 2019 and 2024

In 2019, the proportion of subjects with lower Mc counts in the SCIT group was significantly lower than in the non‐SCIT group (*p *= 0.002) (Figure 3), for example, < 10 Mc, non‐SCIT 30%, SCIT 64%, the Mc count in the 2024 SCIT group was even significantly lower than in the untreated group (*p* = 2.6 × 10^−6^) (Figure 3), for example, < 10 Mc, non‐SCIT 44%, SCIT 90%. In the 2019 SCIT group, the Mc count ranged from 0 to 539 (mean ± standard error: 42.9 ± 21.1), while in the 2024 SCIT group, the cell count ranged from 0 to 22 (2.4 ± 10.9).

**Figure 3:**
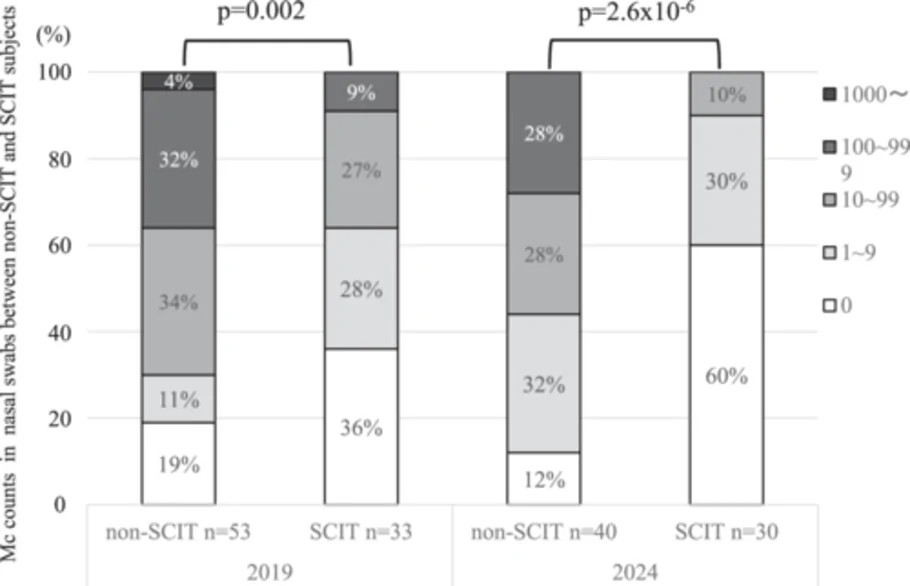
Metachromatic cell (Mc) counts in nasal swabs between non‐SCIT and SCIT subjects in 2019 and 2024. The Mc abundance in subjects of SCIT treatment was significantly lower than that of non‐SCIT subjects in 2019 (*p* = 0.002) and in 2024 (*p* = 2.6 × 10^−6^).

In a 2019 survey, the proportion of SCIT subjects with reduced eosinophils in nasal swabs was greater than in non‐SCIT subjects (*p* = 0.0008), and in 2024, the same was true (*p* = 2.7 × 10^−8^) (Figure 4). There were no significant differences in degrees of neutrophilia in nasal swabs between non‐SCIT and SCIT subjects in 2019 and 2024.

**Figure 4:**
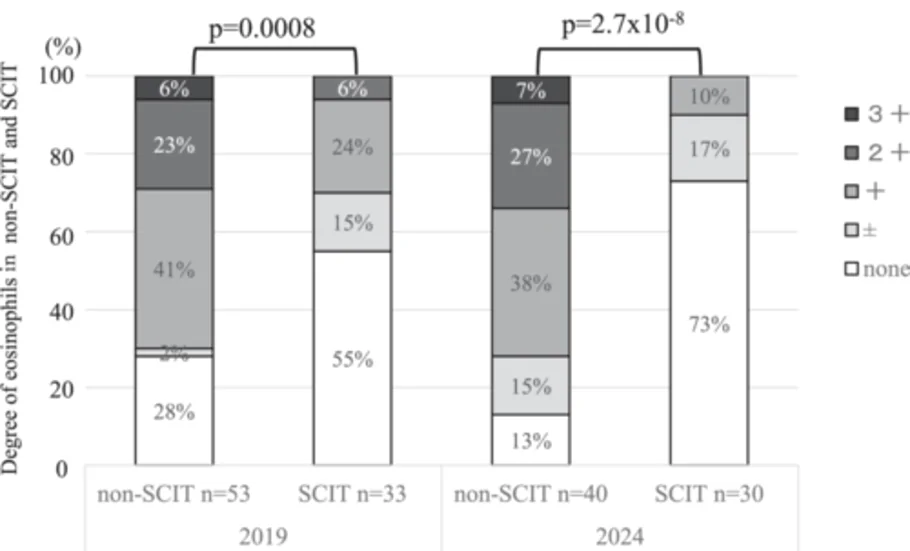
Eosinophils in nasal swabs between non‐SCIT and SCIT subjects in 2019 and 2024. Eosinophils of subjects with SCIT treatment were significantly lower than that of non‐SCIT subjects in 2019 (*p* = 0.0008) and in 2024 (*p* = 2.7 × 10^−8^).

### 3.4 A Study of 13 Cases Who Underwent SCIT Treatment in Both 2019 and 2024

The greater decrease in nasal Mc and eosinophils in 2024 than in 2019 shown above was thought to be largely due to the 13 cases who had continued SCIT for the past 5 years. In these subjects, the allergen dosages of SCIT (subcutaneous immunotherapy) administered between 2014 and 2019 were as follows: 2 subjects received 1 JAU, 2 received 1.4 JAU, 4 received 2 JAU, 4 received 3 JAU, and 1 received 30 JAU; there were no changes in dosage for these 13 subjects over the duration of the study. Furthermore, no adverse events were observed during this period. When the degrees of symptoms, Mc and eosinophil counts in the same 13 patient cases were compared for 2019 and 2024, improvements in these outcome measures were greater given the prolonged treatment with allergen immunotherapy. There was no significant difference in symptom severity between 2019 and 2024 (*p *= 0.2), the number of mast cells (*p *= 0.009) and eosinophils (*p* = 0.003) was significantly lower in 2024. Long‐term treatment with allergen immunotherapy was associated with more marked improvements in these parameters (Figure 5A–D).

**Figure 5:**
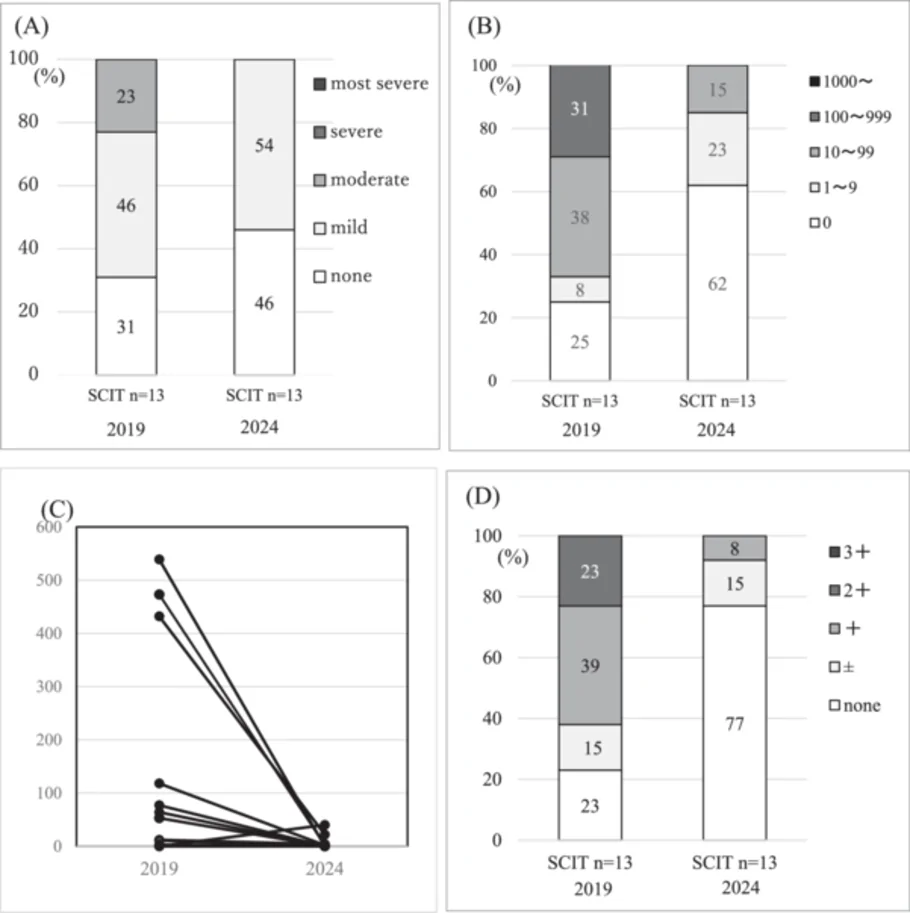
(A) Comparisons of nasal symptom severity between 2019 and 2024 seasons of 13 subjects in both studies (*p* = 0.2). (B) Comparisons of the proportion of the same subjects with Mc in nasal swabs between 2019 and 2024 seasons (*p* = 0.009). (C) Changes in Mc counts in nasal swabs in SCIT from the same subjects assessed during the 2019 and 2024 studies. (D) Comparisons of eosinophil abundance in nasal swabs of the same subjects in both the 2019 and 2024 seasons (*p* = 0.003).

### 3.5 Cost of SLIT and SCIT for Japanese Cedar Pollinosis

We calculated the costs of SLIT and SCIT treatment for the subjects in our clinic with JCP. The average cost of SCIT treatment during the dose optimization phase (~6 months) was ¥53,870 (the subject pays 30%: ¥16160; the remainder is covered by government health insurance). By contrast, the cost of sublingual treatment during the first 6 months is ¥59,950 (¥17,990, paid by the subject). Furthermore, during the maintenance phase, monthly costs are ¥9060 yen (¥2720, paid by the subject) for SLIT, compared to ¥2180 (¥660, paid by the subject) (0.15 mL JAU) for SCIT; in the maintenance phase of treatment, SLIT is significantly more expensive than SCIT.

## 4 Discussion

JCP is common in Japan and SCIT and SLIT are widely used allergen immunotherapies [7, 9, 10, 11, 12, 13]. Our study confirms that with careful allergen dose optimization, SCIT is safe for JCP, and shows that individuals on SCIT for ≥ 5 years had further improvements in symptoms and in the numbers of Mc and eosinophils in nasal swabs.

Local and systemic reactions have been reported with SCIT therapy with various allergens [4, 5]. According to a survey by the AAAAI/ACAAI, many side effects have been reported with SCIT therapy, resulting in 46 deaths between 1945 and 1987 [6, 14]. Causes reportedly included incorrect dosage or concentration, the rush method, and excessively long injection intervals. Furthermore, the use of epinephrine has been recommended, and patients with inadequately controlled asthma or those taking beta‐blockers should be excluded from allergen immunotherapy [5, 15, 16].

Subsequent investigations by AAAAI/ACAAI confirmed two SCIT‐related deaths between 2008 and 2014, and five SCIT‐related deaths between 2015 and 2017 [16]. To prevent not only mortality but also side effects such as rash and respiratory distress, SLIT is recommended as an alternative to SCIT treatment for subjects with JCP, and is currently widely adopted in Japan [10, 11, 12, 13]. However, several studies have shown that SCIT treatment has a lower dropout rate and higher treatment adherence rate than SLIT treatment [17, 18, 19, 20].

Although many physicians administering SCIT for nasal allergies caused by agents such as Japanese cedar pollen have encountered side effects in their patients, there are only two papers reporting on this. One analyzed data regarding Japanese cedar pollen and mite allergens, reporting 17 adverse events between April 1979 and July 1990 [21]. The other paper, published in 2013, noted five cases (0.14%) out of 3481 allergen injections observed over a 2‐year period [22].

Although our clinic did not initially encounter any severe adverse events, cases involving side effects such as systemic skin reactions or asthma attacks were associated with wheal diameters of ≥ 40 mm following SCIT. Consequently, as previously mentioned, we set the threshold for wheal diameter at ≤25 mm, and no serious side effects occurred [7]. Over the 20‐year period since 2006, no serious side effects have been observed in our clinic with SCIT (totaling 42,000 injections using Japanese cedar pollen extract, mite extract, or a mixture of both).

Moreover, a comparison of the costs of SCIT and SLIT using grass pollen shows that SCIT is cheaper than SLIT [23]. However, there are also reports using HD/mite that SLIT is cheaper than SCIT [24]. The difference in these costs is likely due to the duration of the dose optimization and maintenance periods.

When we compared the average costs of SCIT and SLIT in our clinic, costs were similar during the initial 6 months, but SCIT was less expensive than SLIT during the maintenance phase of treatment (after the initial 6 months).

In 2019, administration of a prescribed amount of the pollen extract resulted in improved clinical symptoms and reduced Mc and eosinophil counts in nasal swabs both in‐season and pre‐pollen season compared to the untreated group [7]. In the current study, the 2019 study subjects from in‐season were compared with the 2024 season study. The results showed that the improvement in clinical symptoms was statistically significant in both 2024 and 2019, demonstrating a greater improvement effect in 2024. Furthermore, the reduction in Mc and eosinophil counts in nasal swabs was significantly lower in 2024 compared with 2019 (Figures 1, 2, 3, 4, 5).

Yokohama City conducts an annual survey of Japanese cedar pollen counts using the Durham method, and the pollen count in 2019 was 3696 particles/cm^2^, while in 2024 it was 4432 particles/cm^2^. Therefore, it appears that allergen exposure in 2019 and 2024 was similar or perhaps even greater in 2024, suggesting that allergen exposure cannot explain why the number of these cells in nasal swabs, and clinical symptoms, decreased from 2019 to 2024. Moreover, Figure 1 shows that the optimized maintenance doses established for both the 2019 and 2024 studies were similar.

In two previous SLIT studies [25, 26], the Mc count in nasal swab cytology did not decrease from the time SLIT was initiated until at least 2 years later, whereas in this SCIT study, the Mc count had already decreased within 2 years. Furthermore, even in patients with high Mc and eosinophil counts in nasal swabs in 2019, these counts decreased significantly with continued SCIT treatment for an additional 5 years. Thus, with optimized dosages, SCIT is safe, and many studies have shown that long‐term SCIT improves symptoms. However, we have been unable to identify any other reports that show that long‐term SCIT treatment further reduces Mc and eosinophils in the nasal mucosa.

Other researchers have studied Mc and eosinophils in detail [27, 28, 29, 30, 31]. In particular, mucosal mast cells and eosinophils that increase near the mucosal epithelium are directly affected by the onset of nasal allergy, unlike metachromatic cells and eosinophils in the submucosal connective tissue. This is probably because stem cell factor from epithelial cells reacts with antigens [32], causing chemical mediators to be released from mucosal mast cells, whereas they are not released from connective tissue mast cells, although pretreatment with stem cell factor has shown that release occurs [33].

In a pilot study, we observed that nasal epithelial cells produce an increase in SCF in response to IL4. The increase in Mc and eosinophils in the nasal mucosa epithelial layer may be associated with IL‐4 stimulation of the production of stem cell factor, a growth factor for mast cells produced in the nasal epithelium. We believe that SCIT suppresses IL4 [34, 35, 36] and IL5 [37, 38, 39], thereby decreasing Mc and eosinophils.

In Japan, the trend is currently shifting from SCIT to SLIT, but it may be worth reconsidering the value of SCIT in terms of safety, cost, and compliance.

## Author Contributions

**Otsuka Hirokuni:** conceptualization, methodology, data curation, investigation. **Okubo Kimihiro:** supervision, writing – review and editing. **Otsuka Kuninori:** software, formal analysis. **Gotoh Minoru:** supervision, writing – review and editing.

## Funding

The authors have nothing to report.

## Ethics Statement

Nippon Medical School approved the conduct of this research.

## Conflicts of Interest

The authors declare no conflicts of interest.

## Supporting information


Supporting File

## Data Availability

The data that support the findings of this study are openly available in [repository name e.g “figshare”] at http://doi. org/[doi], reference number [reference number].
