# Supplementary material for: Observational Study on Subcutaneous Immunotherapy for Japanese Cedar Pollinosis in 2024: Comparison With 2019
Source: Immun Inflamm Dis. 2026 Nov 13;14(11):e70589. doi: 10.1002/iid3.70589 (PMC13760727; doi:10.1002/iid3.70589)
Supplement: Supplementary file 1 [file IID3-14-e70589-s001.docx]

STROBE Checklist

I rewrote it as described in the CONSORT Checklist.

This manuscript is based on a cohort study conducted in 2019 and 2024.
